# Association Between the Risk of Dental Caries and *DLX3* Gene Polymorphisms in Chinese Children

**DOI:** 10.3290/j.ohpd.b5779176

**Published:** 2024-10-14

**Authors:** Fang Li, Shusen Xu

**Affiliations:** a Attending Physician, Department of Pediatric Stomatology, Changsha Stomatological Hospital, Changsha, China. Conception and design, acquisition of data, analysis and interpretation of data, wrote the manuscript, read and approved the final manuscript.; b Chief Physician, Department of Pediatric Stomatology, Changsha Stomatological Hospital, Changsha, China. Critically revised the manuscript for important intellectual content, read and approved the final manuscript.

**Keywords:** dental caries, dmft score, DLX3, polymorphisms

## Abstract

**Purpose:**

To explore the association between the risk of dental caries and distal-less homeobox 3 (*DLX3*) gene in Chinese children.

**Materials and Methods:**

Based on the decayed, missing, and filled teeth (dmft) score, the children were divided into a control group (dmft = 0) and a case group (dmft ≥ 1). *DLX3* gene (rs11656951 and rs2278163) polymorphisms were genotyped by polymerase chain reaction (PCR) and Sanger sequencing methods. Possible association of *DLX3* gene (rs11656951 and rs2278163) polymorphisms with dental caries risk was assessed using the chi-squared test. Subgroup analysis of association was assessed by logistic regression analysis for the potential risk factors.

**Results:**

The age at which toothbrushing was started, the brushing frequency, brushing with fluoride toothpaste, and regular dental visits were statistically significantly different between case and control groups (p < 0.05). The frequencies of rs11656951 TT genotype and T allele were statistically significantly higher in the control group than in the case group. The chi-squared test showed that CT genotype (p = 0.026, OR = 0.613, 95%CI = 0.398–0.944) and TT genotype (p = 0.001, OR = 0.378, 95%CI = 0.212–0.673) were negatively correlated with caries susceptibility. The T allele of rs11656951 was more frequently discovered in the control group, and was statistically significantly associated with decreased caries susceptibility (p = 0.001, OR = 0.636, 95%CI = 0.486–0.831). The G allele of rs2278163 was obviously correlated with elevated caries susceptibility (p = 0.049, OR = 1.314, 95%CI = 1.000–1.725). *DLX3* gene rs11656951 TT genotype was a protective factor for caries susceptibility in the subgroups gender, sweets intake, eating before sleep, brushing frequency, brushing with fluoride toothpaste, and dental visits. The GG genotype of rs2278163 was a risk factor for caries in subgroups eating before sleep, brushing without fluoride toothpaste, and regular dental. The TT genotype of rs11656951 was dramatically correlated with reduced caries risk in low (p = 0.004, OR = 0.387, 95%CI = 0.202–0.742) and moderate/high (p = 0.016, OR = 0.360, 95%CI = 0.154–0.840) groups.

**Conclusion:**

*DLX3* gene rs11656951 TT genotype is a protective factor and rs2278163 GG genotype is a risk factor for caries susceptibility, especially in low and moderate/high subgroups.

Dental caries usually occurs in the hard tissues of teeth (dentin and enamel), and is a common chronic oral disease at any age after the eruption of the primary teeth.^6^ Caries results from the interaction of multiple factors, including bacteria, dietary habits, oral hygiene habits, and fluoride use.^4^ The occurrence rate of children’s caries differs between regions.^19^ Although oral health has improved, the prevalence of caries in Chinese children remains high.^10^ Individuals with poor eating habits (sugary foods and drinks), poor oral hygiene, and poor anti-caries predisposition (less saliva, irregular tooth alignment) are susceptible populations for caries.^9,11,18^ Genetic factors define the individual response of the host to environmental factors. Many genes, especially the genes associated with tooth development, have been found to contribute to the development of caries.^8,20^

The distal-less homeobox 3 (*DLX3*) gene belongs to the homeodomain protein family and plays an important role in the development of bones and teeth.^5,14,17,24,25^ DLX3 is the specific substrate for odontoblast differentiation of dental papilla cells (DPCs) in mice.^24^ Zhan et al^23^ suggested that *DLX3* suppresses the proliferation of human DPCs via the Wnt/β-catenin pathway. Mutations of the *DLX3* gene contribute to hypoplasia of dentin and enamel.^22^ rs11656951 is located at a promoter region, and rs2278163 is located at exon1 of the *DLX3* gene; these two polymorphisms might alter the expression or function of *DLX3*. The C allele of rs11656951 was found to be a risk factor for caries development in Brazilian children.^5^ Ohta et al^15^ suggested that rs2278163 T allele was a risk factor for caries in Japanese children. However, the association was has not yet been explored in Chinese children.

Therefore, the current study analysed the association of *DLX3* gene (rs11656951 and rs2278163) polymorphisms with caries risk in Chinese children. In addition, the effects of oral hygiene habits on the association, as well as the association of DLX3 gene polymorphisms with caries severity, were also evaluated in this study.

## MATERIALS AND METHODS

### Subjects

All children ages 2 to 5 years who were found healthy upon examination at Changsha Stomatological Hospital from January 2022 to December 2023 were enrolled in this study. The dental check-up was conducted following World Health Organization criteria.^7^ Children with other dental diseases, systemic diseases, chronic medication use, or who could not cooperate were eliminated from this study.

Baseline data (age, gender), dietary habits (sweets intake, eating before sleep), and oral hygiene habits (age at which toothbrushing was started, brushing frequency, brushing with fluoride toothpaste, and dental visits) were gathered by questionnaire. Questionnaires were filled out by every child’s parents or guardians. Children were divided into a control group (dmft = 0) and a case group (dmft ≥ 1) based on decayed, missing, and filled teeth (dmft) scores. Cases were divided into low (dmft = 1-5), moderate (dmft = 6-9), and high (dmft ≥ 10) severity groups.

The present study was approved by the Ethics Committee of Changsha Stomatological Hospital. All the parents or guardians of children gave their written informed consent.

### Sample Collection

Oral epithelial cells were collected from children by cotton swabs. DNA was isolated from the swabs using a Magnetic Swab DNA Kit (TIANGEN; Beijing, China) according to the manufacturer’s introduction.

DLX3 gene rs11656951 and rs2278163 polymorphisms were amplified by PCR as describe in a previous study.^21^ Then the products were sequenced by the Sanger sequencing method using a 3500 Genetic Analyzer (Applied Biosystems; Foster City, CA, USA).

### Data Analysis

Statistical power was calculated by GPower 3.1 (Heinrich Heine University Dusseldorf, Dusseldorf, Germany). Data analysis was conducted using SPSS 22.0 (IBM; Armonk, NY, USA). Continuous data were analysed using the t-test or non-parametric test, and quantitative data were analysed with the chi-squared test or Fisher’s exact test. The association of *DLX3* gene (rs11656951 and rs2278163) polymorphisms with dcaries risk was assessed by the chi-squared test or Fisher’s exact test. Then the subgroup analysis of association was assessed by logistic regression analysis for the potential risk factors. A statistically significant difference existed when the two tailed p-value was less than 0.05.

## RESULTS

### Characteristics of Participants

A total of 441 children (242 boys and 199 girls) aged from 2 to 5 years participated in this study. The children were divided into a case group (dmft ≥ 1) and a control group (dmft = 0). The control group contained 224 children (123 boys and 101 girls). Give a two-tailed effect size of 0.3, α = 0.05, the statistical power was 0.882. The mean age of the case group was 3.40 ± 1.02 years, and mean age of the control group was 3.35 ± 1.01 years. No statistically significant difference was discovered in age and gender between case and control groups (Table 1, p < 0.05). Dietary habits (sweets intake and eating before sleep) also made no statistically significant difference (p < 0.05). Toothbrushing started at ages over 24 months was statistically significantly more frequently discovered in the case group (p = 0.003). Brushing frequency more than once per day was statistically significantly lower in children with caries (p = 0.004). Children brushing with fluoride toothpaste (p = 0.025) and visiting the dentist regularly (p = 0.001) were statistically significantly more frequently observed in the control group. The mean dmft score was 4.76 ± 3.41 for cases: 70.97% (154/217) of cases had low dmft scores, 22.12% (48/217) cases had moderate dmft scores, and the other 15 cases had high dmft scores (Table 1).

**Table 1:** Characteristics of dental caries children

Characteristics	Cases n = 217 (%)	Controls n = 224 (%)	p-value
**Gender**			0.990
Boys	119 (54.84)	123 (54.91)	
Girls	98 (45.16)	101 (45.09)	
Age (years)	3.40 ± 1.02	3.35 ± 1.01	0.618
**Sweets intake (times per day)**			0.050
< 1	137 (63.13)	161 (71.88)	
≥ 1	80 (36.87)	63 (28.13)	
**Eating before sleep**			0.245
Yes	151 (69.59)	167 (74.55)	
No	66 (30.41)	57 (25.45)	
**Toothbrushing started age (month)**			0.003
< 24	54 (24.88)	85 (37.95)	
≥ 24	163 (75.12)	139 (62.05)	
**Brushing frequency (times/day)**			0.004
< 1	82 (37.79)	56 (25.00)	
≥ 1	135 (62.21)	168 (75.00)	
**Brushing with fluoride toothpaste**			0.025
Yes	43 (19.82)	65 (29.02)	
No	174 (80.18)	159 (70.98)	
**Caries status**			
**Dental visits**			0.001
Yes	37 (17.05)	67 (29.91)	
No	180 (92.95)	157 (70.09)	
dmft score	4.76 ± 3.41		
Low (1–5)	154 (70.97)		
Moderate (6–9)	48 (22.12)		
High (≥ 10)	15 (6.91)		

dmft: decayed, missing, filled teeth.

### Association of DLX3 Polymorphisms with Caries Susceptibility

Genotype distributions of *DLX3* gene rs116948495 and rs2278163 polymorphisms were in accord with the Hardy-Weinberg equilibrium (HWE) test in the control group (Table 2, p < 0.05).

**Table 2:** Association of DLX3 polymorphisms with dental caries

	Cases n = 217 (%)	Controls n = 224 (%)	p	OR (95%CI)
**rs11656951**				
CC	80 (36.87)	54 (24.11)	–	–
CT	109 (50.23)	120 (53.57)	0.026	0.613 (0.398–0.944)
TT	28 (12.90)	50 (22.32)	0.001	0.378 (0.212–0.673)
C	269 (61.98)	228 (50.89)	–	–
T	165 (38.02)	220 (49.11)	0.001	0.636 (0.486–0.831)
P_HWE_		0.283		
**rs2278163**				
AA	73 (33.64)	93 (41.52)	–	–
AG	108 (49.77)	105 (46.88)	0.193	1.310 (0.872–1.970)
GG	36 (16.59)	26 (11.61)	0.058	1.764 (0.978–3.183)
A	254 (58.53)	291 (64.69)	–	–
G	180 (41.47)	157 (35.04)	0.049	1.314 (1.000–1.725)
_PHWE_		0.658		

P_HWE_: p–value for Hardy–Weinberg equilibrium test; OR: odds ratio; 95%CI: 95% confidence interval.

Frequencies of rs11656951 TT genotype and T allele were statistically significantly higher in the control group than in the case group. The chi-squared test showed that rs11656951 CT genotype (p = 0.026, OR = 0.613, 95%CI = 0.398–0.944) and TT genotype (p = 0.001, OR = 0.378, 95%CI = 0.212–0.673) were statistically significantly negatively correlated with caries susceptibility when compared with CC genotype. The T allele was more frequently discovered in the control group, and was statistically significantly associated with decreased caries susceptibility (p = 0.001, OR = 0.636, 95%CI = 0.486–0.831) (Table 2).

The GG genotype of rs2278163 was higher in caries patients than in controls; however, the difference was not statistically significant. Moreover, the rs2278163 genotypes had no statistically significant association with caries susceptibility (Table 2, p < 0.05). The G allele of rs2278163 was statistically significantly higher in caries patients (p = 0.049) and correlated with elevated caries susceptibility (OR = 1.314, 95%CI = 1.000–1.725).

### Subgroup Analysis of the Association Between ***DLX3*** ***P***olymorphisms and Caries Susceptibility based on Characteristics

Subgroup analysis based on characteristics was assessed by logistic regression analysis. The results are shown in Table 3.

**Table 3:** Subgroup analysis of association between DLX3 polymorphisms and caries susceptibility based on characteristics

Characteristics	rs11656951				rs2278163			
	CT		TT		AG		GG	
	p	OR (95%CI)	p	OR (95%CI)	p	OR (95%CI)	p	OR (95%CI)
**Gender**								
Boys	0.874	1.049 (0.582–1.892)	0.096	0.515 (0.236–1.124)	0.962	0.986 (0.559–1.740)	0.122	1.921 (0.840–4.393)
Girls	0.001	0.318 (0.161–0.626)	0.002	0.241 (0.098–0.591)	0.073	1.761 (0.949–3.267)	0.411	1.453 (0.596–3.545)
**Sweets intake (times per day)**								
< 1	0.257	0.735 (0.432–1.251)	0.011	0.408 (0.205–0.813)	0.290	1.305 (0.797–2.138)	0.164	1.726 (0.801–3.720)
≥ 1	0.032	0.432 (0.201–0.929)	0.027	0.288 (0.086–0.867)	0.283	1.536 (0.702–3.360)	0.118	2.244 (0.814–6.182)
**Eating before sleep**								
Yes	0.017	0.537 (0.322–0.896)	0.001	0.305 (0.156–0.596)	0.464	1.201 (0.735–1.963)	0.822	1.083 (0.541–2.167)
No	0.887	0.940 (0.401–2.204)	0.542	0.667 (0.182–2.450)	0.155	1.768 (0.806–3.879)	0.004	10.918 (2.175–54.790)
**Toothbrushing started age (month)**								
< 24	0.499	0.756 (0.335–1.703)	0.901	0.933 (0.314–2.774)	0.309	1.479 (0.696–3.141)	0.220	1.893 (0.683–5.250)
≥ 24	0.075	0.618 (0.364–1.049)	0.000	0.266 (0.133–0.533)	0.462	1.209 (0.729–2.006)	0.139	1.776 (0.831–3.797)
**Brushing frequency (times/day)**								
< 1	0.029	0.406 (0.180–0.913)	0.017	0.235 (0.071–0.773)	0.832	0.921 (0.429–1.974)	0.076	2.809 (0.897–8.795)
≥ 1	0.390	0.792 (0.465–1.348)	0.051	0.508 (0.257–1.004)	0.044	1.681 (1.014–2.788)	0.376	1.407 (0.661–2.999)
**Brushing with fluoride toothpaste**								
Yes	0.256	0.585 (0.232–1.474)	0.001	0.085 (0.021–0.344)	0.723	0.853 (0.354–2.054)	0.949	1.052 (0.219–5.067)
No	0.053	0.613 (0.374–1.006)	0.232	0.658 (0.331–1.306)	0.120	1.459 (0.906–2.350)	0.034	2.070 (1.055–4.064)
**Dental visits**								
Yes	0.003	0.201 (0.068–0.590)	0.010	0.170 (0.044–0.660)	0.667	1.240 (0.466–3.297)	0.008	6.267 (1.607–24.434)
No	0.500	0.846 (0.521–1.375)	0.018	0.452 (0.234–0.872)	0.156	1.406 (0.878–2.250)	0.449	1.299 (0.660–2.558)

dmft: decayed, missing, filled teeth; OR: odds ratio; 95%CI: 95% confidence interval. CC genotype is the reference for rs11656951; AA genotype is the reference for rs2278163.

Subgroup analysis based on gender showed that in comparison with the CC genotype, rs11656951 CT and TT genotypes were obviously correlated with decreased caries susceptibility in girls, but not in boys. When compared to the CC genotype, the CT and TT genotypes of rs11656951 were statistically significantly related to reduced caries susceptibility in children with intake of sweets more than once per day. The TT genotype of rs11656951 was negatively correlated with caries susceptibility in children with sweets intake < 1. Subgroup analysis based on eating before sleep showed that the rs11656951 CT and TT genotypes were negatively associated with caries risk in children who ate before bed. Children with rs11656951 CT and TT genotypes demonstrated a statistically significant correlation with a reduced risk of caries compared to CC genotype carriers when brushing frequency was less than once a day. In comparison with rs11656951 CC genotype, the TT genotype was statistically significantly related to decreased caries risk in children brushing with fluoride toothpaste. When compared with rs11656951 CC genotype, children with TT genotype had a statistically significantly lower caries risk in children with or without dental visits. p-values and OR with 95%CI are presented in Table 3.

For the subgroup analysis of association between rs2278163 genotypes and caries susceptibility, we found that rs2278163 genotypes had no statistically significant association with caries susceptibility in the subgroups gender, eating before sleep, and age at which toothbrushing was started (Table 3, p < 0.05). However, the rs2278163 GG genotype was positively related to caries risk in children not eating before sleep. Furthermore, the rs2278163 AG genotype was positively related to caries risk in children with brushing frequency at least once a day. The rs2278163 GG genotype carriers had a statistically significantly higher caries risk than AA genotype carriers in children brushing without fluoride toothpaste. The GG genotype of rs2278163 polymorphism was positively correlated with caries risk in children with dental visits (Table 3). p-values and OR with 95%CI are presented in Table 3.

In short, rs11656951 TT genotype is a protective factor and rs2278163 GG genotype is a risk factor for caries susceptibility in children with different oral hygiene habits.

### Association of ***DLX3*** Polymorphisms with Caries Severity

217 children with caries were divided into low (dmft = 1–5), moderate (dmft = 6–9), and high (dmft ≥ 10) score groups. Then we analysed the association of *DLX3* polymorphisms with caries severity (Table 4).

**Table 4:** Association of *DLX3* polymorphisms with caries severity

	Controls n = 224 (%)	Low (dmft = 1–5)			Moderate/high (dmft ≥ 6)		
		Cases n = 154 (%)	p	OR (95%CI)	Cases n = 63 (%)	p	OR (95%CI)
**rs11656951**							
CC	54 (24.11)	53 (34.42)			27 (42.86)		
CT	120 (53.57)	82 (53.25)	0.132	0.696 (0.434-1.116)	27 (42.86)	0.011	0.450 (0.241-0.839)
TT	50 (22.32)	19 (12.34)	0.004	0.387 (0.202-0.742)	9 (14.28)	0.016	0.360 (0.154-0.840)
C	228 (50.89)	188 (61.04)			81 (64.29)		
T	220 (49.11)	120 (38.96)	0.006	0.662 (0.493-0.888)	45 (35.71)	0.008	0.576 (0.383-0.867)
**rs2278163**							
AA	93 (41.52)	49 (31.82)			24 (38.10)		
AG	105 (46.88)	80 (51.95)	0.109	1.446 (0.920-2.272)	28 (44.44)	0.916	1.033 (0.560-1.907)
GG	26 (11.61)	25 (16.23)	0.067	1.825 (0.954-3.492)	11 (17.46)	0.244	1.639 (0.711-3.781)
A	291 (64.69)	178 (57.79)			76 (60.32)		
G	157 (35.04)	130 (42.21)	0.046	1.354 (1.005-1.824)	50 (39.68)	0.338	1.219 (0.812-1.831)

dmft: decayed, missing, filled teeth; OR: odds ratio; 95%CI: 95% confidence interval.

In comparison with the rs11656951 CC genotype, the rs11656951 TT genotype was statistically significantly correlated with reduced caries risk in the low dmft group (p = 0.004, OR = 0.387, 95%CI = 0.202–0.742) group. In addition, a negative association was discovered between the rs11656951 T allele and caries susceptibility (p = 0.006, OR = 0.662, 95%CI = 0.493–0.888) in the low dmft group. CT and TT genotypes of rs11656951 were all correlated with caries risk in the moderate/high dmft group. The T allele of rs11656951 was also a protective risk factor for caries. When compared to the rs2278163 A allele, the G allele (p = 0.046, OR = 1.354, 95%CI = 1.005–1.824) was statistically significantly positively related to caries susceptibility in the low dmft group, but not in moderate/high group.

## DISCUSSION

It has been confirmed that caries is affected by oral hygiene habits.^1,16^ In this study, age and gender had no statistically significant effect in the comparison of case- and control-group children. Later toothbrushing-inception age, lower brushing frequency, brushing without fluoride toothpaste and lower regular dental visits were more frequently discovered in children with caries than in control children.

The trajectory of caries is impacted by the interactions among bacteria, oral hygiene habits, and genetic factors.^2,3^ *DLX3* impacts the mineralized dental tissues involved in the formation of teeth.^5^ Abnormal expression of *DLX3* may cause individual susceptibility to risk factors. Therefore, mutations in the *DLX3* gene might disrupt the formation of mineralized dental tissues, ultimately resulting in caries. In the current study, we analysed the association between *DLX3* polymorphisms and caries susceptibility. We found that the CT genotype of rs11656951 was statistically significantly correlated with a 0.613-times lower caries susceptibility. The TT genotype of rs11656951 was statistically significantly related to a 0.378-times lower susceptibility of dental caries. The rs11656951 T allele was more frequently observed in the control group and was statistically significantly correlated with reduced caries risk. This is in accordance with previous study by Chisini et al^5^, who indicated that the rs7501477 T allele combined with the rs11656951 C allele could predict a high risk of caries in Brazilian children. The G allele of rs2278163 was positively related to a 1.314-times greater caries risk, while rs2278163 genotypes had no statistically significant association with caries. This agrees with the study performed by Ohta et al^15^, which indicated that the rs2278163 T allele was positively correlated with caries with high mutans Streptococci. However, Kastovsky et al^13^ found that rs2278163 had no statistically significant association with early childhood caries (ECC).

Oral hygiene habits were obviously different between the case and control groups. However, as no previous studies focused on the association between *DLX3* polymorphisms and oral hygiene habits, we conducted subgroup analyses based on them. The results indicated that rs11656951 CT and TT genotypes were statistically significantly related to 0.318- and 0.241-times lower caries susceptibility, respectively, in girls. However, rs11656951 genotypes were not related to caries risk in boys. In contrast, rs2278163 genotypes had no statistically significant association with caries in girls or boys.

When compared to the CC genotype, the rs11656951 CT genotype was statistically significantly correlated with a 0.432-fold lower caries risk in children who ate/drank sweets more than once per day, while the rs11656951 TT genotype was statistically significantly related to a 0.408-fold and 0.288-fold caries risk reduction, respectively, in children with an intake sweets less than once and more than once per day. In contrast, rs2278163 genotypes were not related to the susceptibility for caries in sweets-intake subgroups.

The CT and TT genotypes of rs11656951 polymorphisms were negatively related a to 0.537- and 0.305-fold lower caries risk, respectively, in children who ate before sleeping. Interestingly, the rs2278163 GG genotype was statistically significantly correlated with an 10.918-times higher caries risk in children not eating before sleep.

Children with the rs11656951 CT genotype and TT genotype were statistically significantly related to 0.406- and 0.235-fold lower caries risk than were CC genotype carriers, when brushing frequency was less than once per day. The AG genotype of rs2278163 was statistically significantly correlated with a 1.681-times higher caries risk in children with a brushing frequency at least one time per day.

The TT genotype of rs11656951 was statistically significantly correlated with reduced caries risk in children brushing with fluoride toothpaste. The rs2278163 GG genotype carriers had a 2.070-times higher caries risk in the subgroup brushing without fluoride toothpaste.

Children with the rs11656951 TT genotype had a statistically significantly lower caries risk with or without regular dental visits. In contrast, the rs2278163 GG genotype was statistically significantly correlated with a 6.267-times higher caries risk in the regular dental visits subgroup.

We also analysed the association of *DLX3* polymorphisms with caries severity. The results showed that the rs11656951 TT genotype was statistically significantly related to lower caries risk in low and moderate/high dmft subgroups. But a positive association was discovered between the rs2278163 G allele and caries risk in the low-dmft group. This might be a result of the small sample size in the moderate/high-dmft subgroup.

The current research had some limitations. First, despite 217 cases and 224 controls providing a reasonable sample size, the study may lack sufficient power to detect smaller effect sizes or associations with caries susceptibility in the high-dmtf subgroup analysis. Larger sample sizes could enhance the reliability of the findings. Second, other *DLX3* gene polymorphisms and interactions between genetic and environmental factors were not explored in this study. Third, this study did not examine underlying mechanisms by which the *DLX3* polymorphisms influence caries susceptibility. Finally, the effects of rs11656951 and rs2278163 on the expression and function of *DLX3* were not explored in this or previous studies. Functional studies or additional molecular analyses could provide deeper insights into the observed associations.

## CONCLUSION

The *DLX3* gene rs11656951 TT genotype is a protective factor and the rs2278163 GG genotype is a risk factor for caries susceptibility. A statistically significant association was also discovered in gender, sweets intake, eating before sleep, brushing frequency, brushing with fluoride toothpaste, and dental visits subgroups. The *DLX3* gene rs11656951 TT genotype was correlated with low and moderate/high dmft scores.
